# Pharmacist Remote Review of Medication Prescriptions for Appropriateness in Pediatric Intensive Care Unit

**DOI:** 10.3389/fphar.2016.00243

**Published:** 2016-08-09

**Authors:** Moran Lazaryan, Ibrahim Abu-Kishk, Noa Rosenfeld-Yehoshua, Sofia Berkovitch, Michal Toledano, Iris Reshef, Tal Kanari, Tomer Ziv-Baran, Matitiahu Berkovitch

**Affiliations:** ^1^Pharmacy Department, Assaf Harofeh Medical Center, Zerifin, affiliated to Sackler School of Medicine, Tel-Aviv UniversityTel-Aviv, Israel; ^2^Pediatric Intensive Care Unit, Assaf Harofeh Medical Center, Zerifin, affiliated to Sackler School of Medicine, Tel-Aviv UniversityTel-Aviv, Israel; ^3^Department of Epidemiology and Preventive Medicine, School of Public Health, Sackler School of Medicine, Tel-Aviv UniversityTel-Aviv, Israel; ^4^Clinical Pharmacology Unit, Assaf Harofeh Medical Center, Zerifin, affiliated to Sackler School of Medicine, Tel-Aviv UniversityTel-Aviv, Israel

**Keywords:** medication review, pediatric intensive care, joint commission international, clinical pharmacist

## Abstract

**Background:** One aspect of ordering and prescribing medication is the requirement for a trained professional to review medication orders or prescriptions for appropriateness. In practice, this review process is usually performed by a clinical pharmacist. However, in many medical centers there is a shortage of staff and a pharmacist is not always available.

**Objective:** To determine whether remote review of medication orders by a pharmacist is a plausible method in a pediatric intensive care unit (PICU).

**Methods:** A pharmacist from the pharmacy department reviewed medication orders of patients admitted to our PICU over a 7-month period for appropriateness. A special form for medical orders was filled in and sent to the physician in the PICU, who replied informing whether the recommendation had been accepted. The time spent by the pharmacist for this activity was recorded.

**Results:** The review time for one medical record was 8.9 (95% CI, 6.9–10.9) min. Every additional drug prescribed increased the total review time by 0.8 (95% CI, 0.45–1.11) min. The pharmacist filled in 186 forms on 117 admissions for 109 children. The median review time was 15 (12.8–18.8) and 12 (9–15) min, respectively, for patients with psychiatric-neurologic disorders compared to those without (*p* = 0.032). Usually, a daily workload of 240 min was needed for the pharmacist accompanying the round in contrast to 108 min per day needed to review all the medical records in 95% of the cases. The physician accepted 51.2%, rejected 11.9%, and made no comment on 36.9% of the recommendations.

**Conclusion:** Hospitals facing budget shortages can carry out focused remote reviews of prescriptions by the pharmacist.

## Introduction

Organizations in more than 100 countries are now working with the Joint Commission International (JCI), which aims to improve patient safety and quality of health care by offering education, publications, advisory services and international accreditation and certification. Many medical centers are keen to achieve JCI accreditation and the organization has published standards for hospitals regarding various aspects of patient care (Joint Commission International, 2014, 5th Edn.). One of these sections covers Medication Management and Use, which includes the organization and management of medication, storing, ordering and prescribing, preparing and dispensing, administration and monitoring. One aspect of ordering and prescribing medication is the requirement for a pharmacist, technician or trained professional to review medication orders or prescriptions for appropriateness. This process should be carried out before the medication is administered to the patient and JCI standards say that this should involve evaluating specific categories, namely the appropriateness of the drug, the dose, frequency, route of administration, therapeutic duplication, and drug interactions.

In practice, this review process is usually performed by a clinical pharmacist joining physicians on their rounds (Lucca et al., 2012). This process takes about 220 ± 40 min (mean 240) based on random calculations made in our medical center during the 6 month period prior to the study. However, in many medical centers, as in ours, there is a shortage of staff and a pharmacist is not always available. If no solution can be found, the relevant JCI standards cannot not be met.

Since in our medical center there is a shortage of clinical pharmacists, we carried out remote reviews by a pharmacist located in the pharmacy department and not on the medical ward. The department chosen for this purpose was the Pediatric Intensive care Unit (PICU), as critically ill patients in general (Moyen et al., 2008), and critically ill children in particular (Miller et al., 2007; Zhang et al., 2012), are more prone to medication errors.

The aims of our study were to evaluate the time needed by the pharmacist to remotely review medication orders from the pharmacy and to characterize the pharmacist recommendations and the decisions taken by the physicians.

## Methods

This study was carried out at Assaf Harofeh Medical Center with approval by the local ethics committee (Study number 84/14). Assaf Harofeh is a 900-bed tertiary hospital affiliated to the Sackler School of Medicine, Tel-Aviv University, Israel. The period of the current study lasted from 6 March 2014 until 6 October 2014. The setting was the seven-bed PICU. The medical records of the PICU patients were computerized, except for the medication orders, which were hand-written by the physician on a separate document. Nurses copied the medication orders onto a list on the nurses' sheet and faxed a hard copy of the updated medication list to the pharmacy every morning. An experienced pharmacist reviewed medication orders from the pharmacy department, without attending the PICU, during the pharmacy department opening hours of 8 h a day, 5 days a week. This task was in addition to routine work in the hospital. Software was installed on every computer in the hospital, including the pharmacy, which allowed remote access to the medical records of the PICU patients.

A special form for medication orders, based on the Medication Management and Use chapter of the JCI standards, was constructed and specific categories were examined by the pharmacist. After the pharmacist filled in the form, it was sent to the senior physicians in the PICU, who sent back a detailed response about whether the recommendation had been accepted or not.

The demographics of each patient were recorded, including age, weight, z-score (which is a standard score related to weight), length of stay (LOS) in the hospital and in the PICU, reason for hospitalization and the number of drugs prescribed.

### Description of the interventions

The interventions were divided into specific categories. The pharmacist filled in a checklist, and added details about the relevant reference in the medical literature.

“*Using drugs according to the labeled indication*” was examined by comparing the medical diagnosis to the indication in the Israeli labeling.

“*There are no contra-indications to use the drugs in the patient*” was verified by the physician's leaflet on prescribing information and another evidence-based source, such as Micromedex (Healthcare Series Truven Health Analytics Inc. Micromedex) or Lexicomp's Drug Reference Handbook.

“*Appropriate dosage*” included consideration of special medical conditions such as renal failure.

“*Appropriate frequency*,” “*appropriate route of administration*,” and “*absence of therapeutic duplication*,” were reviewed. Only clinically significant interactions were reported and these were classified as either *major/contraindication* by Micromedex or rated as *D* = *consider therapy modification or X* = *avoid combination* by Lexi-Interact. Additional comments regarding the technical inappropriateness of the medication orders were also documented, such as using forbidden abbreviations in the medical order and missing details.

Another form was only filled in by the pharmacist when there was any change in the medical treatment or a change in the medical condition that required changing the medical treatment. The pharmacist's recommendation was only written and counted once for the same admission, even if the physician rejected the recommendation without explanation.

### Statistical analysis

Categorical variables were reported as numbers and percentages and continuous variables were reported as means and standard deviations (*SD*) or medians and interquartile ranges (IQR). Continuous variables were tested for normal distribution using the Kolmogorov–Smirnov test and Q-Q plots. Categorical variables were compared using the chi-square test or Fisher's exact test and continuous variables by the independent sample *t*-test or the Mann–Whitney test. Correlations between continuous variables were evaluated using Spearman's rank correlation coefficient. We used a univariate and multivariate linear mixed model to evaluate the association between review time and repeated reviews. The Monte Carlo simulation was used to estimate the time needed to review the records of all the patients each day. We used log-normal distribution for the review time of each patient file and triangular distribution for the daily capacity, based on a minimum of one bed, a maximum of four and a mode of four. The simulation was based on 100,000 scenarios. In each Monte Carlo scenario, random sampling was taken from each of the two distributions. A two-tailed *p* < 0.05 was considered statistically significant. Analyses were performed with SPSS version 21 (IBM Corporation, Armonk, New York, USA).

## Results

Patient characteristics are described in Table 1.

**Table 1:** **Characteristics of patients with and without pharmacist recommendations (PRs)^a^,^b^**.

	**Study population *n* = 117**	**Admissions without PRs *n* = 86**	**Admissions with PRs *n* = 31**	***p***
Age (months), median (IQR)^c^	31.5 (5.0–146.8)	29 (3.0–140.5)	35 (22.0–172.0)	0.103
Male, *n* (%)	73 (62.4%)	55 (64.0%)	18 (58.1%)	0.562
**WEIGHT, MEAN (*SD*)^d^**				
Percentile	25.1 (2.9–56.1)	20.9 (2.9–55.8)	32.3 (3.1–65.2)	0.744
Z-score	−0.9 (1.6)	−0.9 (1.6)	−0.8 (1.6)	0.903
**MAIN REASON FOR HOSPITALIZATION, *n* (%)**				
Infectious diseases	36 (30.8%)	25 (29.1%)	11 (35.5%)	0.507
Respiratory disorders	10 (8.5%)	10 (11.6%)	0 (0.0%)	0.061
Post-operative invasive examination	10 (8.5%)	5 (5.8%)	5 (16.1%)	0.127
Fractures/injuries/accidents	19 (16.2%)	15 (17.4%)	4 (12.9%)	0.557
Medicines	3 (2.6%)	3 (3.5%)	0 (0.0%)	0.564
Cardiovascular	5 (4.3%)	5 (5.8%)	0 (0.0%)	0.323
Oncology	1 (0.9%)	1 (1.2%)	0 (0.0%)	> 0.99
Endocrine disorders	3 (2.6%)	3 (3.5%)	0 (0.0%)	0.564
Psychiatric neurologic disorders	15 (12.8%)	7 (8.1%)	8 (25.8%)	**0.024**
Gastrointestinal disorders	10 (8.5%)	9 (10.5%)	1 (3.2%)	0.287
Burns	3 (2.6%)	1 (1.2%)	2 (6.5%)	0.171
Others	2 (1.7%)			
**NUMBER OF DRUGS, *n* (%)**				
1	12 (10.3%)			
2	25 (21.4%)			
3	13 (11.1%)			
4	13 (11.1%)			
5	15 (12.8%)			
6	8 (6.8%)			
7	11 (9.4%)			
≥8	20 (17.1%)			
Number of drugs, median (IQR)	4 (2.0–7.0)	3 (2.0–5.0)	7 (5.0–10.0)	<**0.001**
Total LOS, median (IQR)	6 (3.0–9.0)	5 (3.0–8.0)	8 (6.0–13.0)	**0.005**
PICU LOS, median (IQR)	3 (2.0–5.0)	2 (1.0–4.0)	3 (2.0–8.0)	**0.048**
Review time (minutes), median (IQR)	13 (10.0–15.0)	12 (9.0–15.0)	15 (15.0–21.3)	**0.001**

a *Data refers to the first reviews of 117 admissions (109 patients)*.

b *PR = at least one Pharmacist Recommendation*.

c *IQR = interquartile range*.

d *SD = standard deviation*.

*p = p-values. The p-values in Bold are those who were statistically significant*.

During the study period, 186 review forms were filled in by the pharmacist and sent to the physicians, regarding 117 admissions of 109 patients. There was at least one pharmacist recommendation on 57 (30.6%) forms, with a total of 84 recommendations on all forms. The median age, percentile, and sex of the patients did not differ significantly between those forms that included recommendations and those that did not. However, patients who received recommendations from the pharmacist on their forms were prescribed more drugs and stayed longer in the hospital and in the PICU than patients who did not receive recommendations (Table 1).

The main reason for hospitalization among both groups was infectious disease, accounting for 35.5 and 29.1% of all patients with pharmacist recommendations and patients with no recommendations, respectively. The main reason for hospitalization did not differ significantly between the two groups, except for psychiatric-neurological disorders, which were more frequent in patients with pharmacist recommendations on their forms (25.8 vs. 8.1%, *p* = 0.024).

The association between patient characteristics and review time is presented in Table 2. Review time was recorded in the first reviews of 79 out of 117 admissions. No statistically significant association was found between review time and age (*p* = 0.238), z-score weight (*p* = 0.852), percentile weight (*p* = 0.849), gender (*p* = 0.167), and some main reasons for hospitalization.

**Table 2:** **Association between patient characteristics and review time**.

	**Review time (minutes)**		
	***n* = admissions^a^**	**value**	***p***
Age (months), r^b^_s_	78	0.135	0.238
**WEIGHT, r^b^_s_**			
Z-score weight	78	0.022	0.852
Percentile weight	78	0.022	0.849
Number of drugs, r_s_	79	0.395	<**0.001**
**GENDER, MEDIAN (IQR)**			
Male	49	12.0 (9.0–15.0)	0.167
Female	30	13.0 (10.8–15.5)	
**MAIN REASON FOR HOSPITALIZATION, MEDIAN (IQR)**			
**Infectious diseases**			
Absent	58	12.0 (9.0–15.0)	**0.049**
Present	21	15.0 (10.0–16.0)	
**Respiratory disorders**			
Absent	69	13.0 (10.0–15.0)	0.294
Present	10	11.5 (8.5–13.5)	
**Post-operative invasive examination**			
Absent	73	13.0 (10.0–15.0)	0.148
Present	6	10.0 (8.0–12.8)	
**Fractures/injuries/accidents**			
Absent	66	13.0 (9.8–15.0)	0.846
Present	13	12.0 (10.0–15.0)	
**Medicines**			
Absent	76	13.0 (10.0–15.0)	0.365
Present	3	12.0 (7.0–13.0)	
**Cardiovascular**			
Absent	75	13.0 (10.0–15.0)	0.254
Present	4	10.0 (7.8–13.8)	
**Oncology**			
Absent	78	12.5 (10.0–15.0)	NA
Present	1	13.0 (13.0–13.0)	
**Endocrine disorders**			
Absent	78	12.5 (10.0–15.0)	NA
Present	1	20.0 (20.0–20.0)	
**Psychiatric neurologic disorders**			
Absent	71	12.0 (9.0–15.0)	**0.032**
Present	8	15.0 (12.8–18.8)	
**GI disorders**			
Absent	71	13.0 (10.0–15.0)	0.436
Present	8	11.5 (8.5–13.0)	
**Burns**			
Absent	77	13.0 (10.0–15.0)	NA
Present	2	12.0 (8.0–16.0)	
**PROPER FORM^c^**			
Absent	65	15.0 (15.0–21.3)	**0.001**
Present	14	12.0 (9.0–15.0)	

a Review time was recorded in the first reviews of 79/117 admissions.

b Data was available for 78/79 admissions.

c Refers to forms filled in by the pharmacist with no recommendations regarding the medical treatment.

rs, Spearman's rank correlation coefficient. p = p-values. The p-values in Bold are those who were statistically significant.

However, the pharmacist spent a longer time reviewing medication orders for patients with an infectious disease or psychiatric-neurologic disorder. The median review time per medical record was 15 min (range 10–16) for patients with infectious diseases compared to 12 min (9–15) for patients with no infectious disease (*p* = 0.049). The median review time was 15 min (12.8–18.8) for patients with psychiatric-neurologic disorders compared to 12 min (9–15) for patients with no psychiatric-neurologic disorders (*p* = 0.032; Table 2).

The overall review time for each medical record was 8.9 min (95% CI, 6.9–10.9), and every additional drug prescribed increased the total review time by 0.8 min (95% CI, 0.45–1.11).

The first assessment carried out showed that repeated reviews of the same clinical case took longer than the first review by 0.85 min (95% confidence interval 0.12–1.59, *p* = 0.024). However, after adjustment for the number of drugs prescribed in each review, there was no correlation between the first and subsequent reviews and the review time. Instead, a correlation was found between the number of drugs and the review time.

**Figure 1:**
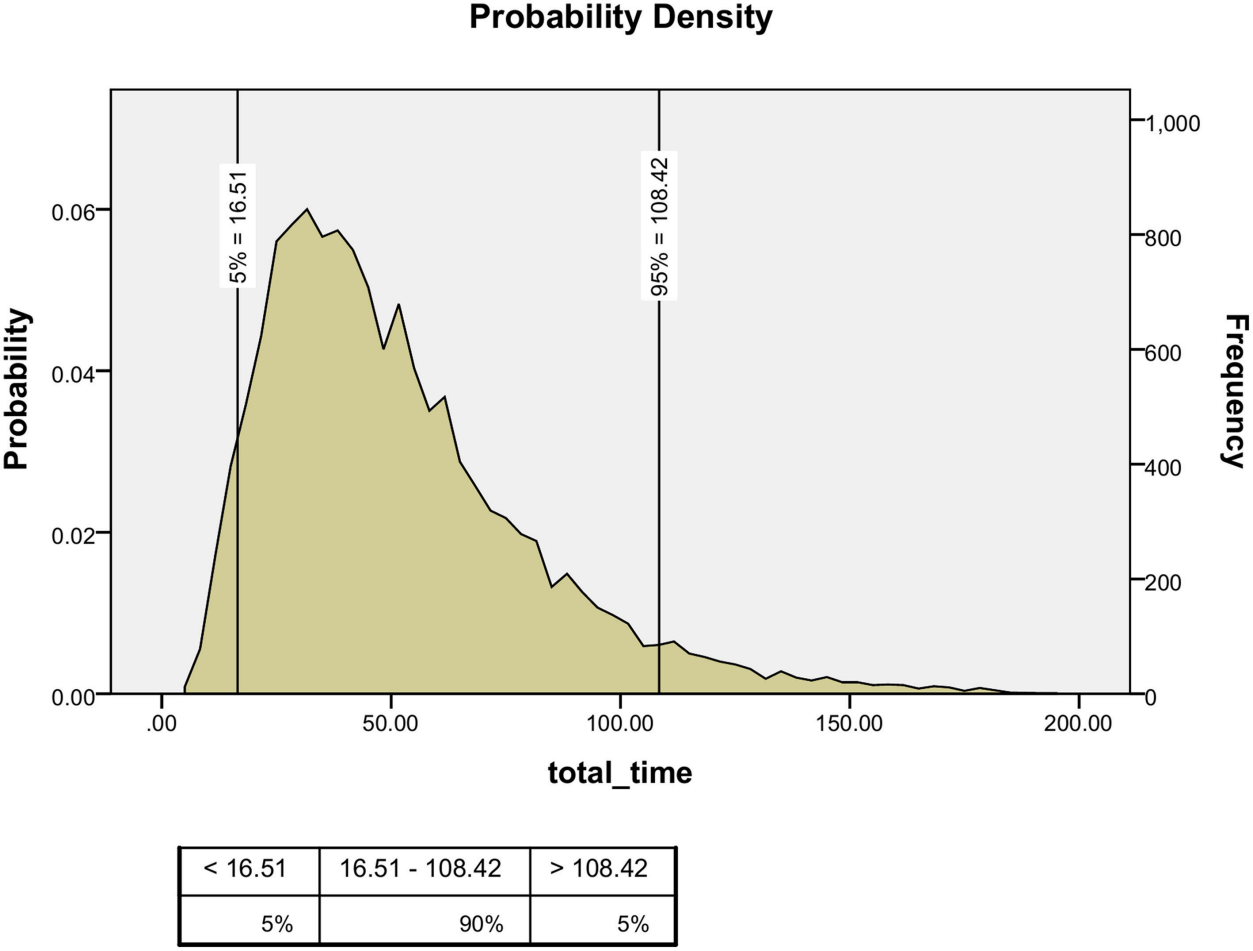
**The estimated daily time needed to review all medication orders in the PICU**.

Using the Monte-Carlo simulation, we estimated that 108 min per day were needed to review all the medical records for this ward in 95% of the cases, assuming that the common daily capacity in the PICU was four of the seven available beds, based on a figure taken from the local computer control unit (Figure 1).

The most common pharmacist recommendations concerned clinically significant interactions, which accounted for 34.5% of all the recommendations, followed by dosage recommendations (26.2%), contraindications (8.3%), drug-indication correlation (3.5%), and frequency as well as route of administration (2.38% each) and other comments (22.6%). There were no recommendations regarding therapeutic duplication (Table 3).

**Table 3:** **Pharmacist recommendations**.

**Pharmacist recommendations**	***n***	**%**
Clinically significant interactions	29	34.52
Dosing recommendations	22	26.19
Additional comments	19	22.62
Contraindication	7	8.33
Drug-indication correlation	3	3.57
Frequency	2	2.38
Route of administration	2	2.38

The physicians accepted 43 (51.2%) of the 84 recommendations from the pharmacist, nine (10.7%) were rejected with an explanation, one (1.2%) was rejected without an explanation and there was no comment on 31 recommendations (36.9%; Table 4).

**Table 4:** **Physician responses to pharmacist recommendations**.

**Pharmacist recommendations**	**Total**	**Accepted**		**Rejected with explanation**		**Rejected with no explanation**		**No comment**	
		***n***	**%**	***n***	**%**	***n***	**%**	***n***	**%**
Drug-indication correlation	3	3.00	100.0	0	0.00	0	0.00	0.00	0.00
Contraindication	7	0.00	0.0	4	57.14	1	14.29	2.00	28.57
Dosing recommendations	22	11.00	50.0	4	18.18	0	0.00	7.00	31.82
Frequency	2	2.00	100.0	0	0.00	0	0.00	0.00	0.00
Route of administration	2	2.00	100.0	0	0.00	0	0.00	0.00	0.00
Therapeutic duplication	0	0.00	0.0	0	0.00	0	0.00	0.00	0.00
Clinically significant interactions	29	15.00	51.7	1	3.45	0	0.00	13.00	44.83
Additional comments	19	10.00	52.6	0	0.00	0	0.00	9.00	47.37

There were 29 recommendations regarding clinically significant drug interactions: in 17 (58.6%) cases the mechanism of the interactions was changes in drug serum concentration, in six (20.7%)—additive QT interval prolongation, in two (6.9%) additive hyperkalemia and in the rest (13.8%) other mechanisms.

There were also 19 additional recommendations made by the pharmacists and 14 (73.7%) of these concerned technical inappropriateness of the medication order, such as unacceptable abbreviations, missing information on route of administration and dosage. A further five (26.3%) concerned inappropriate details of specific terms for pro-re-nata scheduling.

## Discussion

According to the book of standards for hospitals published by the JCI (Joint Commission International, 2014, 5th Edn.), good medication management includes two reviews of each prescription or order. One of the best options is that a clinical pharmacist reviews the medication orders. However, many medical centers cannot afford to employ the number of clinical pharmacists needed, and therefore, are unable to meet the JCI standard.

The role of clinical pharmacists in reducing medication errors has been widely discussed in the literature. Interventions by clinical pharmacists have been shown to improve patient safety, satisfaction and compliance rates as well as decreasing adverse drug events and the length of hospital stay (Leape et al., 1999; Moffett et al., 2008; Makowsky et al., 2009; Klopotowska et al., 2010; Cunningham, 2012; Kelishadi and Mousavinasab, 2012; Zhang et al., 2012; Ho et al., 2013; Jiang et al., 2014; Zhai et al., 2015). Some studies have shown considerable cost savings (Krupicka et al., 2002; Larochelle et al., 2012; Lucca et al., 2012; Michalets et al., 2015).

In the current study, the estimated time needed by an experienced pharmacist, located in the pharmacy, to evaluate the appropriateness of prescriptions requested for a seven-bed PICU, with an average occupancy rate of four beds per day, was 108 min a day. This is in contrast to the same pharmacist being present during the round with an average workload of 240 min per day. Our method of medication assessment may save 45% of the workload and salaries needed for clinical pharmacists.

The review time for one medical record was 8.9 min and every additional drug prescribed increased the review time by 0.8 min (95% CI 0.45–1.11). This time frame enabled the pharmacist to perform other activities in the pharmacy.

In our study, the pharmacist made 84 recommendations on 117 admissions of 109 patients, with 0.71 pharmacist recommendations per admission and 0.77 per patient. The most common recommendations concerned clinically significant interactions and dosage recommendations. Critically ill patients are often prescribed a large number of drugs with an increased potential for drug interactions. Children are more prone to medication errors (Wong et al., 2004; Miller et al., 2007), mostly because the dosage is usually calculated based on age and weight, in contrast to adults, where most drugs are usually prescribed as a fixed dose.

Patients with pharmacist recommendations in our study were prescribed more medications and had a longer total hospital stay and a longer stay in the PICU. This may indicate that patients requiring intervention from the pharmacist were more severely ill. Other characteristics of the patients with recommendations, in comparison to those without, have been mentioned in other studies (Krupicka et al., 2002; Moffett et al., 2008). These characteristics may help hospitals with budget shortages to prioritize patients who require pharmacist involvement.

Fourteen (16.6%) of the 84 recommendations in our study concerned technical inappropriateness of the medical order, such as using unaccepted abbreviations or missing details of the route of administration. These recommendations could have been prevented had the medication order been computerized rather than hand-written. Shulman et al. found that the total proportion of medication errors was significantly lower with computerized physician order entry (CPOE) than with hand-written prescriptions (4.8 vs. 6.7%, respectively, *p* < 0.04; Shulman et al., 2005). Azizeh et al. found that using CPOE with standardized concentrations decreased dispensing errors on continuous infusion medications and required less processing time than hand-written prescriptions (Azizeh et al., 2010).

We assumed that remote reviewing of specific categories of medical orders would take less time than that usually spent by the clinical pharmacist on the ward. However, comparing the time needed to review medical orders as reported in other studies was problematic, because pharmacist intervention was not uniform. The reported time was usually the routine daily time spent by the pharmacist in the ward, rather than the time needed for reviewing one medical record.

Krupicka et al. investigated the impact of a pediatric clinical pharmacist in the 10-bed PICU (Krupicka et al., 2002). The pharmacist made 172 recommendations for a total for 77 patients, with 2.23 recommendations per patient. It was reported that patients with recommendations from the pharmacist had a statistically significant longer length of stay in the PICU. The most common pharmacist interventions reported were dosage recommendations and drug information. The average time spent by the clinical pharmacist in the PICU was 0.73 h per day.

Larochelle et al. described an 8-month review of clinical pharmacist interventions in an 18-bed tertiary care PICU (Larochelle et al., 2012). They reported an average of 5.5 interventions per patient, with dosage recommendations and pharmacokinetics being the most common type of interventions. The pharmacist was available for patient care rounds and follow-up visits in the afternoon for 8 h a day for ~12–15 days each month.

The number of pharmacy recommendations per patient in our study, which was 0.77, seemed lower than the numbers reported in other studies. This could be explained by the fact that the pharmacist focused on specific categories of drug dispensing, rather than widespread counseling to the medical staff. However, reviewing specific categories in the medical treatment, as required by the JCI, is more practical than a comprehensive review of medical orders, especially in hospitals that have a shortage of pharmacists.

The physicians accepted 51.2% of pharmacist recommendations, with 10.7% being rejected and in the rest there was no comment. It is important to mention that we classified a physician's comment as a “rejection” when the physician did not change the medicinal treatment despite of the pharmacist note.

This includes two situations. Firstly, in cases of clinically significant drug-drug interactions, which are frequently inevitable in the context of PICU patients. Even though the comments in such cases were classified as a rejection, the physician was usually unaware of the existence of such interaction and was grateful for the information. Secondly, we also considered a comment as a “rejection” when the physician kept prescribing a medicine despite a contraindication to its use in certain conditions. Yet, it is important to mention that the Israeli prescribing information frequently mentions more contraindications than in the accepted literature. Moreover, the pharmacist in our study carried out remote reviews, which could explain the high percentage of recommendations that the physicians did not comment on. We assume that when the pharmacist is physically present in the ward, the contact with the physician is closer, compared to electronic or paper communication. A recent meta-analysis described eight key elements reflecting effective collaboration between a pharmacist and a general practitioner (GP). One of them included face-to-face meetings to discuss drug-related problems. The meta-analysis showed a significant association between these key elements of the intervention and the number of recommendations implemented (Kwint et al., 2013).

The limitations of this study include the relatively small number of the participants, the fact that the severity of the illness was not taken into consideration by the pharmacist and, the lack of direct contact with the patient and his physician, which may be the reason why some pharmacist recommendations were rejected by the physicians. Yet the study's significance lies in offering a method to assess medications where there is a shortage of clinical pharmacists.

## Conclusions

In order to meet JCI standards there is a need for a pharmacist review of the medication orders or prescriptions for appropriateness. This time consuming process requires budgets that are not always available. The current study proposes a time saving method by showing that the assessment of medication orders remotely by a pharmacist makes a considerable contribution to the medical treatment. Hospitals facing budget shortfalls can carry out focused remote reviews of prescriptions by a pharmacist.

## Author contributions

ML, MB, IA, and NR conceptualized and designed the study, carried out the analyses, drafted the initial manuscript, and approved the final manuscript as submitted. SB and MT designed the study, drafted the initial manuscript, and approved the final manuscript as submitted. IR and TK carried out the analyses, drafted the initial manuscript, and approved the final manuscript as submitted.

## Author note

The study was presented at the European Society of Developmental Perinatal and Pediatric Pharmacology (ESDPPP) conference, June 2015, Belgrade, Serbia.

### Conflict of interest statement

The authors declare that the research was conducted in the absence of any commercial or financial relationships that could be construed as a potential conflict of interest.
